# Two-year clinical follow-up of the Multicenter Randomized Clinical Trial of Endovascular Treatment for Acute Ischemic Stroke in The Netherlands (MR CLEAN): design and statistical analysis plan of the extended follow-up study

**DOI:** 10.1186/s13063-016-1669-6

**Published:** 2016-11-22

**Authors:** Lucie A. van den Berg, Marcel G. W. Dijkgraaf, Olvert A. Berkhemer, Puck S. S. Fransen, Debbie Beumer, Hester Lingsma, Charles B. M. Majoie, Diederik W. J. Dippel, Aad J. van der Lugt, Robert J. van Oostenbrugge, Wim H. van Zwam, Yvo B. W. E. M. Roos, for the MR CLEAN invesigators

**Affiliations:** 1 Department of Neurology, Academic Medical Center, PO Box 22660, 1100 DD Amsterdam, The Netherlands; 2 Clinical Research Unit of the Academic Medical Center, PO Box 22660, 1100 DD Amsterdam, The Netherlands; 3 Department of Radiology, Academic Medical Center, PO Box 22660, 1100 DD Amsterdam, The Netherlands; 4 Department of Neurology, Erasmus MC University Medical Center Rotterdam, PO Box 2040, 3000 CA Rotterdam, The Netherlands; 5 Department of Neurology, Maastricht University Medical Center, PO Box 5800, 6202 AZ Maastricht, The Netherlands; 6 Department of Public Health, Erasmus MC University Medical Center, Rotterdam, The Netherlands; 7 Department of Radiology, Erasmus MC University Medical Center, PO Box 2040, 3000 CA Rotterdam, The Netherlands; 8 Department of Radiology, Maastricht University Medical Center, PO Box 5800, 6202 AZ Maastricht, The Netherlands

**Keywords:** Intravenous thrombolysis, Endovascular treatment, Acute ischemic stroke, Randomized controlled trial, Mechanical thrombectomy

## Abstract

**Background:**

MR CLEAN was the first randomized trial to demonstrate the short-term clinical effectiveness of endovascular treatment in patients with acute ischemic stroke caused by large vessel occlusion in the anterior circulation. Several other trials confirmed that endovascular treatment improves clinical outcome at three months. However, limited data are available on long-term clinical outcome. We aimed to estimate the effect of endovascular treatment on functional outcome at two-year follow-up in patients with acute ischemic stroke. Secondly, we aimed to assess the effect of endovascular treatment on major vascular events and mortality during two years of follow-up.

**Methods:**

MR CLEAN is a multicenter clinical trial with randomized treatment allocation, open-label treatment, and blinded endpoint evaluation. Patients included were 18 years or older with acute ischemic stroke caused by a proven anterior proximal artery occlusion who could be treated within six hours after stroke onset. The intervention contrast was endovascular treatment and usual care versus no endovascular treatment and usual care. The current study extended the follow-up duration from three months to two years.

The primary outcome is the score on the modified Rankin scale at two years. Secondary outcomes include all-cause mortality and the occurrence of major vascular events within two years of follow-up.

**Discussion:**

The results of our study provide information on the long-term clinical effectiveness of endovascular treatment, which may have implications for individual treatment decisions and estimates of cost-effectiveness.

**Trial registration:**

NTR1804. Registered on 7 May 2009; ISRCTN10888758. Registered on 24 July 2012 (main MR CLEAN trial); NTR5073. Registered on 26 February 2015 (extended follow-up study).

## Introduction

Stroke is one of the leading causes of disability and death worldwide [1]. Until recently the only proven effective therapy for acute ischemic stroke was intravenous (IV) thrombolysis with recombinant tissue plasminogen activator (rt-PA) [2]. In January 2015 the results of the Multicenter Randomized Clinical Trial of Endovascular Treatment for Acute Ischemic Stroke in the Netherlands (MR CLEAN) were published and demonstrated the clinical effectiveness of endovascular treatment with respect to functional recovery at three months [3]. In the subsequent months several other trials confirmed these results [4–8]. However, results on long-term clinical outcome are still lacking. In the current paper we present the design and statistical analysis plan (SAP) of the extended, two-year clinical follow-up study of the MR CLEAN trial. The primary objective of our study is to estimate the effect of endovascular treatment in comparison to standard treatment on functional outcome at two-year follow-up in patients with acute ischemic stroke caused by a proximal occlusion in the anterior cerebral circulation. Secondary objectives include the effect of endovascular treatment on major vascular events, mortality, and quality of life during two years of follow-up.

## Methods

### Study design and overall study plan

MR CLEAN was a multicenter clinical trial with randomized treatment allocation, open-label treatment, and blinded endpoint evaluation. The intervention contrast was endovascular treatment (mechanical thrombectomy with stent retriever in 97% of patients) versus no endovascular treatment. The treatment was provided in addition to usual care, which included intravenously administered rt-PA (in approximately 90% of patients). Patients were randomized in a 1:1 ratio. The randomization procedure was web-based, with the use of permuted blocks, stratified by center, with dichotomized score on the National Institutes of Health Stroke Scale (NIHSS), treatment with intravenously administered rt-PA, and intended mechanical treatment [9]. Data were collected at baseline, 24 hours, one week, and three months for the main trial. Detailed information on the main trial, treatment, blinding, statistical analysis, and determination of sample size is given in the protocol and SAP of the MR CLEAN trial [10].

Because of funding issues, the extended follow-up study came into effect only in May 2013. At that moment the inclusion of the MR CLEAN trial was well halfway of the projected 500 patients to be included. As a result, many patients had already completed their three-month follow-up, and some patients had even passed the two-year follow-up point. After checking the Dutch Death Certificate Register, surviving patients were re-invited to take part in the extended follow-up study. If patients did not wish to participate, a reply was to be sent back to the trial office. Approximately two weeks after sending the invitation letter, all willing participants were contacted by telephone to confirm their participation and to explain additional study goals and activities. For the remaining MR CLEAN trial inclusions and their legal representatives, the new follow-up duration including additional study activities was explained in an adjusted MR CLEAN informed consent letter. All participating patients or their primary caregivers, for patients who were unable to respond, were contacted by telephone at six months, one year, 18 months and two years of follow-up. One experienced research nurse, blinded for treatment allocation, assessed functional outcome using the modified Rankin scale (mRS) and noted the occurrence of medical events in between follow-ups by a telephone interview [11]. The patient or his/her primary caregiver was also invited to complete the EuroQol  EQ-5D-3L questionnaire to assess quality of life [12].

### Study population

Patients aged 18 years or older with acute ischemic stroke caused by an anterior proximal artery occlusion who were able to be treated within six hours after stroke onset were eligible for inclusion in MR CLEAN. Detailed inclusion and exclusion criteria are described in the protocol of the MR CLEAN trial [10].

Because the current extended follow-up study started more than two years later than the main MR CLEAN trial, many patients had already completed their participation in the main trial at the start of the extended follow-up study. As a result different groups of patients emerge:

1. Patients randomized before May 2011 (group 1)
2. Patients randomized between May 2011 and May 2013 who:
  -  Had died by May 2013 (group 2)
  -  Were untraceable or were living abroad in May 2013 (group 3)
  -  Did not provide consent for extended follow-up or who withdrew consent during follow-up (group 4)
  -  Provided consent for extended follow-up study with subsequently a different number of measurements during follow-up (group 5)
3. Patients randomized after May 2013 (group 6).

As a consequence of the above, the population for the two-year follow-up analysis will be somewhat smaller than the one for the three-month follow-up analysis, with a varying number of available measurements over time. All patients with an available two-year follow-up visit will be included in the main long-term follow-up analyses. These include patients from group 2, group 5 and group 6.  One major concern regarding the estimation of treatment effect in this selected patient population might be an unbalanced distribution in treatment arms at two years. A possible threat for the treatment effect estimation at two years could be bias created by patients who decided not to consent for the long-term follow-up study (group 4). One reason for not participating in the long-term follow-up study might be that these patients had worse outcomes after three months and therefore felt they were not capable of participating in the follow-up visits. They may also be dissatisfied with being allocated to standard treatment as a reason for refusal for further participation. Both motivations may lead to relatively more patients available for the long-term follow-up who received intervention and had a good outcome at three months. This may cause serious selection bias, resulting in overestimation of the treatment effect.

To assess whether indeed the patient selection resulted in an unbalanced distribution, and thus an unrepresentative two-year trial population, we will compare main prognostic variables, three-month functional outcome, and treatment allocation between patients who did not consent (group 4) to patients who consented for the extended follow-up study (groups 5 and 6) and perform additional sensitivity analyses [13–15]. In addition we will compare treatment effect on functional outcome at three months in patients who did not consent for the extended follow-up to patients with available two-year follow-up.

#### Subgroup populations

The effect of intervention on the main endpoint, the mRS score at two years of follow-up, will be analyzed in the same predefined subgroups as in the main trial, including the following:

- Age 80 or older at time of randomization versus age younger than 80 at time of randomization
- NIHSS at randomization in tertiles (2–15, 16–19, and 20 or higher)
- Terminal internal carotid artery occlusion present versus no terminal internal carotid artery occlusion
- Time since stroke onset to randomization 120 minutes or less versus more than 120 minutes
- Extracranial >50% carotid stenosis or occlusion versus no >50% carotid stenosis or occlusion
- Alberta Stroke Program Early Computed Tomography Score (ASPECTS) 0–4, 5–7, 8–10

### Study endpoints

#### Primary outcome measure

The primary outcome is the clinical outcome on the mRS at two years. The mRS is an ordinal scale ranging from 0 (no disability) to 6 (death) [11].

#### Secondary outcome measures

Secondary outcome measures include:

- All-cause mortality within two years of follow-up. In addition, we will assess mortality in the period between three months and two years after inclusion in patients who were alive at three months of follow-up.
- Improvement according to the classical dichotomizations of the mRS at two years including mRS 0–1 (excellent outcome) versus 2–6; mRS 0–2 (independency) versus 3–6, and mRS 0–3 (moderate good outcome) versus 4–6.
- First new major vascular events between three months and two years of follow-up.
- The quality of life using the EuroQol EQ-5D-3L questionnaire.

#### Definitions and assessment of major vascular events

##### Definition of major vascular event

Major vascular events include fatal or nonfatal cardiac events, fatal or nonfatal stroke, or fatal or nonfatal major peripheral arterial or thrombo-embolic events. Cardiac events include myocardial infarction, resuscitation after cardiac arrest, and hospitalization for unstable angina or cardiac insufficiency. Major peripheral events include all events related to noncoronary arterial disease leading to hospitalization or revascularization (e.g., new or worsening of claudication leading to revascularization). Major thrombo-embolic events include pulmonary embolism or cerebral venous thrombosis.

Recurrent stroke was defined according to the World Health Organization criteria as “rapidly developing symptoms and/or signs of focal, and at times global, loss of cerebral function, with symptoms lasting more than 24 hours or leading to death with no apparent cause other than that of vascular origin” [16]. We defined a recurrent stroke as a stroke, using the above definition, in which (1) there was clinical evidence of the sudden onset of a new focal neurological deficit with no apparent cause other than that of vascular origin (i.e., the deficit could not be ascribed to an intercurrent acute illness, epileptic seizure, or toxic effect) occurring at any time after the index stroke; or (2) there was clinical evidence of the sudden onset of an exacerbation of a previous focal neurological deficit with no apparent cause other than that of vascular origin [17, 18].

##### Assessment of major vascular events

Reported events were checked by contacting the treating physicians, hospitals, and/or general practitioners. Events had to be confirmed by available reports of events or otherwise orally confirmed by the treating physician or general practitioner. All events were centrally reviewed by two investigators (LAvdB and YBWEMR), who were blinded for treatment allocation.

### Statistical analysis

#### General considerations

LAvdB, MGWD, and YBWEMR will perform all analyses. Estimates of treatment effects will be presented with 95% confidence intervals, unless specified otherwise. A two-tailed *P* value of ≤0.05 will be considered significant for all measures. All analyses will be based on the intention-to-treat principle. Analyses of the efficacy parameters will be adjusted according to the SAP of the main trial, and results of the unadjusted analyses will be provided. The analysis will be performed after the last randomized patient has reached the two year follow-up, after all data have been validated and the database is cleaned and after approval of the SAP by the executive committee.

As of 30 March 2016, follow-up was completed. The database for the long-term clinical follow-up was frozen as of 6 April 2016. The same day the data were extracted from the online database. On 1 May 2016 the SAP was finalized and agreed on by the executive committee for the unblinded analyses of the two-year results by LAvdB, MGWD, and YBWEMR. Preliminary results were presented at the 2016 European Stroke Organization Conference, held on 12 May 2016 in Barcelona, Spain.

#### Analysis of demographics and baseline characteristics

The baseline characteristics of all subjects listed per treatment sequence will be outlined in a table and summarized with descriptive statistics.

#### Analyses of efficacy parameters

##### Primary endpoint

The long-term follow-up data will be analyzed at two years using the same methods as the three-month follow-up in MR CLEAN. The primary effect parameter takes the whole range of the mRS into account and will be estimated as an odds ratio for improvement on the mRS by ordinal logistic regression (shift analysis) [19].

Multivariable regression analysis will be used to adjust for chance imbalances in main prognostic variables between intervention and control group in the primary effect analysis, but also in all secondary analyses and subgroup analyses [20]. These main prognostic variables are age, stroke severity (NIHSS) at baseline, time to randomization, previous stroke, atrial fibrillation, diabetes mellitus, and terminal internal carotid artery occlusion versus no terminal internal carotid artery occlusion.

##### Secondary endpoints

All-cause mortality for both time periods (inclusion until two years, and between three months and two years) will be analyzed using the log-rank test with Kaplan-Meier plots. To adjust for the prespecified factors, a Cox regression model will be applied with a risk ratio expressed as a hazard ratio.

Major vascular events between three months and two years of follow-up will be analyzed by using person-years at risk to calculate the event rate in both treatment arms. This way events reported by patients who were lost to follow-up during the extended follow-up will be taken into account. Between-group differences will be expressed as relative risk reduction.

The dichotomizations of the mRS at two years including mRS 0–1 versus 2–6 and mRS 0–2 versus 3–6 will be estimated with a multiple logistic regression with the odds ratio as effect parameter.

Quality of life will be displayed graphically per treatment group and for each dimension of the EQ-5D: mobility, self-care, usual activities, pain/complaints, and mood (anxiety/depression). Furthermore, a composite health utility value will be derived for each completed five-dimensional EQ-5D questionnaire with readily available scoring algorithms, reflecting societal preferences for different health states, elicited by time trade-off techniques applied to the general population [21]. The effect parameter will be a regression parameter beta, estimated with a multiple linear regression model.

### Additional procedures and sensitivity analyses for missing data

As stated earlier, patients who did not consent to the extended follow-up may introduce an important selection bias. To gain as much information as (legally) possible on the clinical status of these patients, a waiver from the Institutional Review Board was obtained to assess the vital status of these patients at two years of follow-up. This information will be used for the survival analysis as well as for the sensitivity analyses of the primary outcome.

First we will compare patients who did not consent to patients who consented to the extended follow-up, including the following variables: main prognostic variables, treatment allocation, and functional outcome at three months (dichotomized mRS 0–3 versus 4–6). To test for any statistically significant differences between groups, categorical variables will be compared by the chi-square test and continuous variables by Student’s *t* test or, in case of a non-normal distribution, the Mann-Whitney U test. Finally, we will develop alternative modeling scenarios to assess the robustness of the base case results (complete case analyses) of the primary outcome. Based on clinically plausible scenarios, the analyses will consist of two different scenarios created by single imputation for the mRS at two years in patients who did not consent:

1. Last observation carried forward: patients of whom we have information on vital status at two years and who died will be scored as an mRS 6 (death), and for all other patients the mRS score will be imputed with the available three month mRS score.
2. Worst case scenario: patients of whom we have information on vital status at two years and who died will be scored as an mRS 6; for patients with an mRS score of 5 (=severe disability) at three months, the mRS score will be imputed with an mRS 5; and for patients with an mRS <5 at three months, the mRS score will be imputed with mRS 5.

Finally, we will compare treatment effect on functional outcome at three months in patients who did not consent to the extended follow-up to patients with available two-year follow-up.

### Subgroup analyses

Subgroups will be analyzed with an interaction term for each subgroup by treatment allocation and reported as subgroup-specific estimates with 95% confidence intervals, displayed in a forest plot.

## Discussion

The MR CLEAN trial aimed to evaluate the effect of endovascular treatment on functional outcome after three months in patients with acute ischemic stroke. Additionally, it assessed safety and effect on recanalization of endovascular therapy. Limited evidence is available for the long-term outcome after endovascular treatment for acute ischemic stroke. In the current study we extend the follow-up duration to two years after randomization to estimate the effect of endovascular treatment on functional outcome over the longer term. Secondary objectives include the effect of endovascular treatment on major vascular events and mortality during two years. This paper allows for peer review of the proposed methods and provides a transparent statement of the planned analyses.

### Limitations and concerns

The sample size of the main trial was not powered for an extended follow-up. During longer term follow-up studies, loss to follow-up is a well-known phenomenon, resulting in smaller sample sizes. Furthermore, loss to follow-up may cause serious attrition bias. Both these problems may play an important role in our study, mainly because of the late start of the extended follow-up study. We therefore will provide clear information on the flow of patients through the study and differences in baseline and measured variables according to provision of consent for the long-term follow-up, and we will perform additional sensitivity analyses for different scenarios.

The results of our study will provide information on the long-term clinical effectiveness of endovascular treatment for patients with acute ischemic stroke. The benefit of endovascular treatment on short-term disability might translate into longer term improvements in survival and functional status, which could influence individual treatment decisions and estimates of cost-effectiveness. Subsequently, it will have important additional value concerning implementation of endovascular treatment all over the world.

## Appendix

### List of MR CLEAN investigators and affiliations

Olvert A. Berkhemer, M.D.,^1,2^ Puck S.S. Fransen, M.D.,^2,3^ Debbie Beumer, M.D.^2,4^ Lucie A. van den Berg, M.D.,^5^ Hester F. Lingsma, M.D., Ph.D.,^7^ Albert J. Yoo, M.D.,^8^ Wouter J. Schonewille, M.D.,^9^ Jan Albert Vos, M.D., Ph.D.,^10^ Paul J. Nederkoorn, M.D., Ph.D.,^5^ Marieke J.H. Wermer, M.D., Ph.D.,^11^ Marianne A.A. van Walderveen, M.D., Ph.D.,^12^ Julie Staals, M.D., Ph.D.,^4^ Jeannette Hofmeijer, M.D., Ph.D.,^13^ Jacques A. van Oostayen, M.D., Ph.D.,^14^ Geert J. Lycklama à Nijeholt, M.D., Ph.D.,^15^ Jelis Boiten, M.D., Ph.D.,^16^ Patrick A. Brouwer, M.D.,^3^ Bart J. Emmer, M.D., Ph.D.,^3^ Sebastiaan F. de Bruijn, M.D., Ph.D.,^17^ Lukas C. van Dijk, M.D.,^18^ L. Jaap Kappelle, M.D., Ph.D.,^19^ Rob H. Lo, M.D.,^20^ Ewoud J. van Dijk, M.D., Ph.D.,^21^ Joost de Vries, M.D., Ph.D.,^22^ Paul L.M. de Kort, M.D., Ph.D.,^23^ Willem Jan J. van Rooij, M.D., Ph.D.,^24^ Jan S.P. van den Berg, M.D., Ph.D.,^25^ Boudewijn A.A.M. van Hasselt, M.D.,^26^ Leo A.M. Aerden, M.D., Ph.D.,^27^ René J. Dallinga, M.D.,^28^ Marieke C. Visser, M.D., Ph.D.,^29^ Joseph C.J. Bot, M.D., Ph.D.,^30^ Patrick C. Vroomen, M.D., Ph.D.,^31^ Omid Eshghi, M.D., ^32^ Tobien H.C.M.L. Schreuder, M.D.,^33^ Roel J.J. Heijboer, M.D.,^34^ Koos Keizer, M.D., Ph.D.,^35^ Alexander V. Tielbeek, M.D., Ph.D.,^36^ Heleen M. den Hertog, M.D., Ph.D.,^37^ Dick G. Gerrits, M.D., ^38^ Renske M. van den Berg-Vos, M.D., Ph.D.,^39^ Giorgos B. Karas, M.D.,^40^ Ewout W. Steyerberg, M.D., Ph.D.,^7^ H. Zwenneke Flach, M.D.,^26^ Henk A. Marquering Ph.D.,^41,1^ Marieke E.S. Sprengers, M.D., Ph.D.,^1^ Sjoerd F.M. Jenniskens, M.D., Ph.D.,^42^ Ludo F.M. Beenen, M.D.,^1^ René van den Berg, M.D., Ph.D.,^1^ Peter J. Koudstaal, M.D., Ph.D.,^2^ Wim H. van Zwam, M.D., Ph.D.,^6^ Yvo B.W.E.M. Roos, M.D., Ph.D., ^5^ Aad van der Lugt, M.D., Ph.D., ^3^ Robert J. van Oostenbrugge, M.D., Ph.D., ^4^ Charles B.L.M. Majoie, M.D., Ph.D., ^1^ and Diederik W.J. Dippel, M.D., Ph.D.^2^

1 Department of Radiology, Academic Medical Center Amsterdam, the Netherlands; 2 Department of Neurology, Erasmus MC University Medical Center Rotterdam, the Netherlands; 3 Department of Radiology, Erasmus MC University Medical Center Rotterdam, the Netherlands; 4 Department of Neurology, Maastricht University Medical Center and Cardiovascular Research Institute Maastricht (CARIM), the Netherlands; 5 Department of Neurology, Academic Medical Center Amsterdam, the Netherlands; 6 Department of Radiology, Maastricht University Medical Center, the Netherlands; 7 Department of Public Health, Erasmus MC University Medical Center Rotterdam, the Netherlands; 8 Department of Radiology, Texas Stroke Institute, Texas, United States of America; 9 Department of Neurology, Sint Antonius Hospital, Nieuwegein, the Netherlands; 10 Department of Radiology, Sint Antonius Hospital, Nieuwegein, the Netherlands; 11 Department of Neurology, Leiden University Medical Center, the Netherlands; 12 Department of Radiology, Leiden University Medical Center, the Netherlands; 13 Department of Neurology, Rijnstate Hospital, Arnhem, the Netherlands; 14 Department of Radiology, Rijnstate Hospital, Arnhem, the Netherlands; 15 Department of Radiology, MC Haaglanden, the Hague, the Netherlands; 16 Department of Neurology, MC Haaglanden, the Hague, the Netherlands; 17 Department of Neurology, HAGA Hospital, the Hague, the Netherlands; 18 Department of Radiology, HAGA Hospital, the Hague, the Netherlands; 19 Department of Neurology, University Medical Center Utrecht, the Netherlands; 20 Department of Radiology, University Medical Center Utrecht, the Netherlands; 21 Department of Neurology, Radboud University Medical Center, Nijmegen, the Netherlands; 22 Department of Neurosurgery, Radboud University Medical Center, Nijmegen, the Netherlands; 23 Department of Neurology, Sint Elisabeth Hospital, Tilburg, the Netherlands; 24 Department of Radiology, Sint Elisabeth Hospital, Tilburg, the Netherlands; 25 Department of Neurology, Isala Klinieken, Zwolle, the Netherlands; 26 Department of Radiology, Isala Klinieken, Zwolle, the Netherlands; 27 Department of Neurology, Reinier de Graaf Gasthuis, Delft, the Netherlands; 28 Department of Radiology, Reinier de Graaf Gasthuis, Delft, the Netherlands; 29 Department of Neurology, VU Medical Center, Amsterdam, the Netherlands; 30 Department of Radiology, VU Medical Center, Amsterdam, the Netherlands; 31 Department of Neurology, University Medical Center Groningen, the Netherlands; 32 Department of Radiology, University Medical Center Groningen, the Netherlands; 33 Department of Neurology, Atrium Medical Center, Heerlen, the Netherlands; 34 Department of Radiology, Atrium Medical Center, Heerlen, the Netherlands; 35 Department of Neurology, Catharina Hospital, Eindhoven, the Netherlands; 36 Department of Radiology, Catharina Hospital, Eindhoven, the Netherlands 37 Department of Neurology, Medical Spectrum Twente, Enschede, the Netherlands; 38 Department of Radiology, Medical Spectrum Twente, Enschede, the Netherlands; 39 Department of Neurology, Sint Lucas Andreas Hospital, Amsterdam, the Netherlands; 40 Department of Radiology, Sint Lucas Andreas Hospital, Amsterdam, the Netherlands; 41 Department of Biomedical Engineering and Physics, Academic Medical Center Amsterdam, the Netherlands; 42 Department of Radiology, Radboud University Medical Center, Nijmegen, the Netherlands

## Abbreviations

ASPECTS: Alberta Stroke Program Early Computed Tomography Score
EQ-5D: EuroQol 5D measurement tool for health-related quality of life
IV: Intravenous
MR CLEAN: Multicenter Randomized Clinical trial of Endovascular treatment for Acute Ischemic Stroke in the Netherlands
mRS: Modified Rankin scale
NIHSS: National Institutes of Health Stroke Scale
rt-PA: recombinant tissue plasminogen activator
SAP: Statistical analysis plan
